# P-462. Temporal Clustering of Mycoplasma pneumoniae–Associated Encephalitis and Stroke in Children: A Multicenter Study During a Post-COVID-19 Epidemic

**DOI:** 10.1093/ofid/ofaf695.677

**Published:** 2026-01-11

**Authors:** Seung Ha Song, Dayun Kang, Ye Kyung Kim, Woo Joong Kim, Ki Wook Yun

**Affiliations:** Department of Pediatrics, SMG-SNU Boramae Medical Center, Seoul, Seoul-t'ukpyolsi, Republic of Korea; Department of Pediatrics, Seoul National University Children’s Hospital, Seoul, Seoul-t'ukpyolsi, Republic of Korea; Seoul National University Children's Hospital, Seoul, Seoul-t'ukpyolsi, Republic of Korea; Seoul National University Children's Hospital, Seoul, Seoul-t'ukpyolsi, Republic of Korea; Seoul National University Children's Hospital, Seoul, Seoul-t'ukpyolsi, Republic of Korea

## Abstract

**Background:**

*Mycoplasma pneumoniae* (MP) commonly causes respiratory infections and CNS complications, including encephalitis and ischemic stroke in children. During a 2023–2024 MP epidemic in South Korea, a temporal increase in pediatric CNS complications was noted.

**Methods:**

We conducted a retrospective study of children (≤18 years) hospitalized with MP-associated encephalitis or stroke across three hospitals in South Korea (October 2023–December 2024). MP infection was defined by positive serology and/or PCR. Encephalitis was diagnosed based on acute altered mental status with supportive cerebrospinal fluid, neuroimaging, or EEG findings. Stroke was diagnosed by focal neurologic deficits with corresponding ischemic lesions on neuroimaging.

**Results:**

Seventeen patients (median age 8.8 years [IQR 7.8–11.3]; 53% male) were included, comprising 12 encephalitis and 5 stroke cases. All occurred in 2024, with 94% clustered between June and December, coinciding with a regional MP surge. CNS manifestations occurred in 2.2% (17/775) of children diagnosed with MP infection.

Stroke patients showed focal deficits (100%) and headache (40%), while encephalitis patients exhibited altered mental status (92%) and seizures (33%). The median interval from neurologic symptom onset to hospital visit was longer in stroke cases (8.0 days [IQR 5.3–9.3]) than in encephalitis cases (2.5 days [IQR 2.0–5.3]; *p* = 0.390).

All stroke patients showed infarctions involving the right middle cerebral artery territory on MRI. Multifocal T2/FLAIR hyperintensities were observed in 58% of encephalitis cases.

Corticosteroids were administered to 2 of 5 stroke and 7 of 12 encephalitis patients; IVIG was used in 4 encephalitis cases. Neurologic sequelae occurred in 41% overall (stroke 60%, encephalitis 33%), and no deaths were reported.

**Conclusion:**

During Korea’s first post-COVID-19 MP epidemic, MP-associated pediatric CNS complications clustered temporally and demonstrated distinct clinical and radiologic profiles. These findings highlight the need for early neurologic assessment in children with MP infection, particularly during epidemic periods.

**Disclosures:**

All Authors: No reported disclosures

